# Gut health, stress, and immunity in neonatal dairy calves: the host side of host-pathogen interactions

**DOI:** 10.1186/s40104-020-00509-3

**Published:** 2020-11-09

**Authors:** Johan. S. Osorio

**Affiliations:** Dairy and Food Science Department, South Dakota State University, 113 H Alfred Dairy Science Hall, Brookings, SD 57007 USA

**Keywords:** Dairy calves, Fecal RNA, Neonatal diarrhea

## Abstract

The cumulative evidence that perinatal events have long-lasting ripple effects through the life of livestock animals should impact future nutritional and management recommendations at the farm level. The implications of fetal programming due to malnutrition, including neonatal survival and lower birth weights, have been characterized, particularly during early and mid-gestation, when placental and early fetal stages are being developed. The accelerated fetal growth during late pregnancy has been known for some time, while the impact of maternal stressors during this time on fetal development and by extent its postnatal repercussions on health and performance are still being defined. Maternal stressors during late pregnancy cannot only influence colostrogenesis but also compromise adequate intestinal development in the fetus, thus, that further limits the newborn’s ability to absorb nutrients, bioactive compounds, and immunity (i.e., immunoglobulins, cytokines, and immune cells) from colostrum. These negative effects set the newborn calf to a challenging start in life by compromising passive immunity and intestinal maturation needed to establish a mature postnatal mucosal immune system while needing to digest and absorb nutrients in milk or milk replacer. Besides the dense-nutrient content and immunity in colostrum, it contains bioactive compounds such as growth factors, hormones, and cholesterol as well as molecular signals or instructions [e.g., microRNAs (miRNAs) and long non-coding RNAs (lncRNAs)] transferred from mother to offspring with the aim to influence postnatal gut maturation. The recent change in paradigm regarding prenatal materno-fetal microbiota inoculation and likely the presence of microbiota in the developing fetus intestine needs to be addressed in future research in ruminants. There still much to know on what prenatal or postnatal factors may predispose neonates to become susceptible to enteropathogens (e.g., enterotoxigenic *Escherichia coli*), causing diarrhea. From the host-side of this host-pathogen interaction, molecular data such as fecal RNA could, over time, help fill those gaps in knowledge. In addition, merging this novel fecal RNA approach with more established microbiome techniques can provide a more holistic picture of an enteropathogenesis and potentially uncover control points that can be addressed through management or nutrition at the farm level to minimize preweaning morbidity and mortality.

## Introduction

During gestation, maternal interaction with the environment influences the growth and development of the fetus before it is exposed to more direct influences in an extrauterine environment. For this extrauterine adaptation, the dam produces nutrient-dense colostrum with the aim to equip the offspring with the necessary biological arsenal, including immunoglobulins and other bioactive compounds, with the intent to confer a transient immunity while stimulating gut maturation.

In ruminants, the maternal influence on fetal development and postnatal performance of offspring is well known [1]. The impact of specific nutritional factors such as energy [2] and amino acids (AA) [3] has been established, together with environmental factors, such as heat stress [4]. In terms of nutrition, competition for nutrient requirements between the fetus and dam has been well recognized [5], where fetal requirements take priority towards the end of gestation. Therefore, maternal nutrition during late gestation in dairy cows has been reported to have a significant impact on fetal development and postnatal performance. For instance, greater energy intake in late gestation can affect birth weight and neonatal immunocompetence [6]. Similar effects related to improvements on the immune system have been observed in offspring born to dams supplemented with adequate supplementation of trace minerals [7] and AA [8, 9] during late pregnancy in dairy cows.

Colostrum is the first extrauterine source of nutrients, and a temporary immunization delivered system to the newborn calf, therefore, it has a long-lasting impact throughout life [10]. These effects have been widely recognized in livestock science, and in dairy cows, it has been reflected in the large amount of colostrum research conducted since the late 1970s [11]. Interestingly, less is known on the impact of late gestation maternal nutrition on colostrogenesis and colostrum quality. In turn, this may affect the newborn ability to adapt to the new extrauterine environment [10]. In addition to nutrients and immunoglobulins, the colostrum contains various bioactive compounds such as insulin-like growth factors (IGF), insulin, and cholesterol [12] or molecular signals in the form of microRNA (miRNA) [13] and long non-coding RNA (lncRNA) [14] that interact or stimulate developmental programs in naïve enterocytes in the newborn calf. These interactions or stimuli are a major turning point for adequate maturation of the enterocyte lining, promoting the establishment of intestinal digestion and absorption [15].

The newborn calf needs to adapt to a completely new environment with a high pathogen load, must thermoregulate its own body, must change its digestive system from umbilical nutrient delivery during gestation to digesting bottle- or bucket-fed milk, and then must transition from pre-ruminant to ruminant. This scenario puts a tremendous amount of stress on the newborn calf, and likely its immunological status will be tested within the first 3 weeks of life, when harmful pathogens including enterotoxigenic *Escherichia coli* (ETEC), coronavirus, and *Cryptosporidium* spp. colonize the digestive tract [16]. This coincides with the need to transition from a passive immunity (i.e., colostrum) to an active immunity [17]. Interestingly, the pathology of diarrhea can be traced back to initial interactions between opportunistic pathogens and enterocytes. For instance, ETEC can anchor themselves to specific cell membrane glycolipids in enterocytes [18]. Once fixed to the intestinal epithelium, ETEC can transfer small peptides or enterotoxins, which cause an intracellular signal cascade culminating in rapid efflux of electrolytes (i.e., Cl^−^) from enterocytes into the intestinal lumen, causing dehydration and diarrhea [19].

A further in-depth exploration into the biology of enterocytes in neonatal dairy calves is warranted to overcome the ever-growing challenges of raising replacement heifers in the dairy industry. This exploration will advance our understanding of the biology of enterocytes in newborns and will help delineate a clearer picture of how bioactive compounds in colostrum may affect the enterocyte maturation or how opportunistic pathogens may insert cellular programs into enterocytes to pave their way to conquer the enterocyte lining. Therefore, the objective of this review is to underscore the importance of studying how stress and immunity can affect neonatal enterocyte biology in the context of ruminants and propose alternative methods to obtain crucial biological information from enterocytes in neonates.

### Maternal influence and stressors on intestinal development

There is a large body of evidence on the maternal influence on fetal development and its postnatal consequences. In ruminants, particular effects of maternal stressors such as malnutrition [20] and thermal stress [4] or common prepartal nutritional strategies [21] on fetal development have been reviewed or evaluated previously. They have provided the initial steps to understand the degree of the impact of these effects on postnatal development and performance in the young ruminant. However, the precise implications of these maternal stressors’ effects on intestinal development and subsequent postnatal maturation are only beginning to be understood [22]. A summary of the effects of these stressors, management practices, and other factors on intestinal development is provided in Table 1.

**Table 1:** The impact of perinatal stressors or conditions on indicators of intestinal development or maturation

Stressor or effect^a^	Species or model^b^	Effect on intestinal development^c^	Age measured, d	Reference
IUGR	Sheep	↓ Intestinal mass and length ↓ Crypt depth and mucosal size	90 and 140 d of gestation	[23, 24]
Nutrient restriction	Cow	↓ Villus and crypt density ↑ Duodenal upregulation of *SLC5A1*, *CD36*, and *CCK*	135 d postnatal	[25]
IUGR	Sheep	↓ Villus height and width	90 d of gestation	[23]
Nutrient restriction	Sheep	↓ Jejunum vascularity and *GUCY1B3*	135 d of gestation	[26]
Overnutrition	Sheep	↑ Jejunal hyperplasia and vascularity	20 and 180 d postnatal	[27, 28]
Heat stress	Cow	↓ Passive immune transfer due to reduced intestinal surface area	1 and 2 d postnatal	[29]
Negative DCAD diet	Cow	↓ Colostrum IgG absorption	1–2 d postnatal	[32]
Negative DCAD diet	Cow	↔ Colostrum IgG absorption	1 d postnatal	[21]
Injected cortisol	Rabbit	↑ Brush border enzymes and Na^+^/K^+^ ATPase	10–12 d postnatal	[33]
Cortisol	H4 cells	↑ Gene related to cell polarity, tight junction formation, and interactions with extracellular matrices	–	[34]
Cortisol	H4 cells	↓ Attenuates proinflammatory insults	–	[35]
miRNAs	Mouse and IPEC-J2 cells	↑ Villus height and crypt depth	–	[36]

^a^IUGR = Intrauterine growth retardation; DCAD = dietary cation-anion difference; miRNAs = microRNAs (~ 18–25 nucleotides)

^b^H4 cells = human fetal small intestinal epithelial cell line; IPEC-J2 = porcine small intestinal epithelial cell line

^c^↑ = increase; ↓ = decrease; ↔ = no effect

Meyer and Caton [22] reviewed the impact of IUGR on intestinal development across several species, and while IUGR during early- and mid-gestation does not seem to affect intestinal mass, IUGR in mid- and late gestation did reduce intestinal mass. Besides mass and length, functional aspects of the intestinal development in the fetus seem to be more susceptible to negative effects of IUGR, including decreased villus and crypt density [25], villus height and width [23], crypt depth [23, 24], and mucosal size [23, 24]. Meyer and Caton [22] also compared the effects of the IUGR model with less drastic nutrient restriction models on intestinal development (60% nutrient requirements). Similar to IUGR, nutrient restriction during mid- to late pregnancy in ewes seemed to have a more pronounced effect on intestinal development than nutrient restriction during early to mid-pregnancy [22]. In ruminants, less is known about the prenatal molecular adaptations in the developing gastrointestinal tract product of IUGR or nutrient restrictions. However, it appears that transcriptional alterations related to nitric oxide vasodilation and angiogenesis, including *GUCY1B3* (soluble guanylate cyclase 1 β3), are highly responsive to maternal undernutrition [22]. Neville et al. [26] used a nutrient restriction model (60% nutrient requirements) in sheep during late gestation and observed a reduction in fetal jejunum vascularity concomitantly with a downregulation of *GUCY1B3*. Later, Meyer et al. [37] observed an upregulation of *GUCY1B3* in intestinal samples from steers at 450 d of age, which were born to dams that were subjected to a nutrition restriction (70% energy requirements) while supplemented with a rumen-undegradable protein. More recently, da Cruz et al. [25] observed an upregulation in mRNA expression of cell membrane transporters and neuropeptides including *SLC5A1* (Solute carrier family 5 member 1), *CD36* (Cluster of differentiation 36), and *CCK* (cholecystokinin) in duodenal samples collected from 135 days old steers born to dams offered a restricted diet (75% protein requirements) during mid to late pregnancy. In the same study, it was observed a lower birth body weight (BW) in offspring born to dams fed the  protein restricted diet, but this effect on BW disappeared by weaning. These effects on gene expression underscore the long-term effects of maternal nutrition on intestinal development and maturation. In addition, maternal protein undernutrition led to upregulation of genes related to glucose and long-chain fatty acids transporters in the offspring as potential coping mechanisms to improve nutrient absorption while upregulating *CCK* a known neuropeptide secreted by duodenal cells and responsible for inhibiting satiety and increase nutrient absorption [25]. It could be speculated that a disrupted intestinal development may set in place an alternative long-term cellular programing to allow the offspring to compensate for maternal stressors.

Nutritional requirements decrease as dairy cows enter late lactation and early dry period, which generally leads to a positive energy balance, and as a consequence, a significant portion of dairy cows get overconditioned during this period [38]. In recent years, this scenario has become arguably more prevalent across commercial dairy farms [20]. In terms of fetal development, maternal overcondition during mid to late gestation can cause undesirable alterations in the offspring, including metabolic syndrome [39]. In contrast to maternal undernutrition and the IUGR model, maternal overnutrition, and its effects on fetal programming in cattle has received less attention among animal scientists. As a result, there are very few studies on maternal overnutrition effects on intestinal development. Meyer and Caton [22] compiled the effects of maternal overnutrition during mid and late gestation on small intestine growth and development in postnatal lambs at 20 and 180 d of age, and they observed that, in general, overnurishment did not affect small intestine mass growth, but caused jejunal hyperplasia and vascularity [27, 28]. Important to notice is the fact that these effects were consistent in lambs at 20 and 180 d of age, and lambs were fed a common artificial colostrum and milk replacer, which confirms that prenatal overnutrition effects will persist well into life.

Thermal stress during pregnancy exerts carryover effects on fetal development and postnatal health and performance [4]. However, there is a lack of data on the impact of thermal stress during pregnancy on intestinal ontogeny and development. Recent studies suggest that maternal heat stress may compromise passive immune transfer from colostrum due to impaired intestinal absorption or reduced intestinal surface area regardless of colostrum quality [29, 30]. On the other hand, the effects of cold stress during pregnancy are scarcer than heat stress. However, research using rodent models suggests that prenatal cold stress can modulate the maternal hypothalamus-pituitary-adrenocortical (HPA) activity [40] responsible for releasing glucocorticoids, including cortisol, as well as altering maternal metabolism [41]. Interestingly, maternal cold stress can affect placental physiology [42]; therefore, it is conceivable that temperature is likely to affect intestinal development, similar to other maternal stressors such as undernutrition [43].

Another potential maternal influence in intestinal development could be the common practice to feed negative dietary cation-anion difference (DCAD) diets to close-up cows in most commercial dairy farms. This practice is prevalent based on consistent results on lowering the risks for clinical hypocalcemia, retained placenta, metritis, and overall diseases during early postpartum [44]. Early studies showed a potential for a negative effect of maternal acidified diets (+ 75 vs. + 445 mEq/kg) on colostrum IgG absorption in the offspring [32], while others did not observe any effects on plasma mineral content of offspring when dams were fed acidified diets (− 30 vs. + 90 mEq/kg) [45]. More recent data suggest that perhaps the length of time under a negative DCAD diet prepartum (12 vs. 42 d) might have a greater influence on the offspring’s health and birth BW than just DCAD level itself [21]. Collazos et al. [21] observed a reduced gestation length when feeding negative DCAD diets for 42 d prepartum, which could explain the lower birth BW and reduced blood neutrophil and lymphocyte concentration in offspring in this group. However, Collazos et al. [21] did not observe any adverse effects of DCAD diets on colostrum IgG absorption. Abuelo [20] concluded that the effects of dams receiving a negative DCAD diet during close-up are transient in nature and do not affect passive transfer of immunity and, by extension, the health and performance of the neonate. In the context of intestinal development, there is no data available on the effects of prepartal DCAD diets on intestinal ontogeny and development and how this might affect the long-term performance and feed efficiency of heifers during their first lactation.

### Postnatal challenges in neonates affect intestinal maturation

Parturition is the culmination of the gestation, and it is often considered as the most stressful event in life for both the dam and the newborn. Therefore, is it not a coincidence that cortisol, the stress hormone, is highly relevant during parturition. Maternal cortisol commonly surges in the days leading to parturition as a biological queue to start this process, while fetal cortisol increases over the weeks prior to calving [17]. The release of cortisol or glucocorticoids, in general, is highly dependent on HPA activity, an endocrine mechanism regulated by inflammatory stimuli such as pro-inflammatory cytokines, prostaglandins, apoptotic cells, among others [17].

The effects of fetal cortisol in neonatal calf immunity and stress have been reviewed previously [17]; and, Hulbert and Moisa [17] described that calves that experienced dystocia had low levels of glucocorticoids rather than high levels, despite the association of glucocorticoids with stress. Also, they further interpreted that glucocorticoids are needed in the perinatal calf for various functions, including gut health since glucocorticoids stimulate tight junction and mucosal formation [17]. Glucocorticoid effects in gut health have been explored over several studies [46], yielding additional mechanisms for an intestinal maturation mediated by glucocorticoids, including increase brush border enzymes such as sucrase and alkaline phosphate and cell membrane transporters such as Na^+^/K^+^ ATPase [33]. From a molecular standpoint, glucocorticoids exert their effects through their cognate cytosolic receptor, the glucocorticoid receptor, a transcription factor that upon activation can bind DNA with the aim to regulate the transcription of genes related to development, metabolism, and immune response [47]. Regarding neonatal gut health, however, gene regulation by the glucocorticoid receptor has been associated with maturation of the intestinal epithelium [34, 35]. Under normal conditions, perinatal cortisol dictates many biological programs, including those related to tissue maturation in the neonate, and data suggest that additional stressors around calving can compromise neonate tissue responsiveness, including intestinal response to the normal perinatal surge in cortisol levels [34].

The differential thermoneutral zone between dam and offspring has been known for some time [48], and, in ruminants, this knowledge has been combined with data on heat stress physiology which have given rise to several publications on the overall impact of maternal heat stress on newborn calf development and performance [4]. However, less is known about the effect of heat stress on postnatal intestinal maturation [4]. In this context, there are hints in the literature suggesting an effect of heat stress on postnatal intestinal maturation; however, these are commonly confounded with maternal heat stress effects on prenatal intestinal development. Therefore, from this perspective, experiments designed to isolate the pre- and postnatal effects of heat stress abatement in neonates such as Dado-Senn et al. [31], are essential to elucidate the early life effects of heat stress on intestinal maturation in neonates. Dado-Senn et al. [31] reported a positive postnatal cooling effect on the neonatal incidence of fever, infections, and overall medications regardless of prenatal heat stress conditions. In the same study, greater plasma IgG was observed in calves born to dams under a prepartal cooling treatment consisting of shaded area plus fans and water soakers. However, the effect of postnatal cooling treatment (i.e., shaded area plus fans) on plasma IgG levels within 24 h after birth was not evaluated. However, these data highlight the importance of mitigating early life stressors such as heat stress, and at the same time, warrant the need for further exploration of the extent of these positive effects related to postnatal intestinal maturation.

### Colostrum effects on postnatal intestinal maturation

Colostrum intake is not only essential for transfer of passive immunity to the offspring but also contains nutrients and bioactive compounds such as growth factors, hormones, and cholesterol or molecular signals in the form of miRNA [49] or lncRNA [14]. These bioactive compounds interact or stimulate biological programs in the naïve or immature enterocyte lining of newborns causing an intestinal maturation [15]. This was evident in Yang et al. [50], where a noticeable morphological improvement in the small intestine was observed in calves fed colostrum in comparison to calves fed transitional milk (i.e., 2–3 d after calving) or bulk tank milk. Those morphological improvements included better villus length and width, crypt depth, villus height to crypt depth ratio, and mucosal thickness. Intestinal maturation is fundamental for the neonate to develop a functional gastrointestinal tract capable of digest and absorb nutrients in milk or milk replacer while protecting the neonate against opportunistic pathogens.

The various bioactive compounds in colostrum drive morphological and transcriptional alterations in the maturing intestinal lining of neonatal calves, and these alterations have been previously reviewed [15]. Colostrum is a more nutrient-dense fluid than regular milk, and this also applies to most bioactive compounds in colostrum, where IGF, growth hormone (GH), and insulin are found at a higher concentration in colostrum than milk [15]. These bioactive compounds commonly bind to specific cell membrane receptors in neonatal enterocytes. This binding effect creates an intracellular signal cascade that is usually integrated through phosphorylation activity via kinase proteins such as mitogen-activated protein kinase (MAPK) or Janus kinase (JAK). This signal cascade culminates in the activation of transcription factors such as the family of signal transducer and activator of transcription proteins (STATs). The transcription factors such as STATs or glucocorticoid receptor (explained previously) are the ultimate biological relays that are in charge to carry on the upstream signal cascade [e.g., GH-GH receptor (GHR)-STAT axis], which results in the transcriptional activation of target genes by binding to a specific sequence in the DNA promoter region of such genes. The cellular response to the bioactive compounds in colostrum are embedded in these transcriptional alterations, which encode for proteins that will enforce an activation of cell growth, proliferation, energy metabolism, and immune function [15].

The specific mechanisms of how bioactive compounds in colostrum orchestrate and promote intestinal maturation in neonates are complex. However, it has been reported that cholesterol can activate the intestinal ATP-binding cassette transporter A1 (ABCA1), a cholesterol transporter and consequently enhancing intestinal lipid absorption [15]. This is crucial at this point since neonates will rely on lipids as one of the primary energy sources. The signal cascade downstream of ABCA1 is partially mediated by sequential phosphorylation of the JAK2-STAT axis [51].

Upon activation by GH, GHR may stimulate one of its canonical cellular signals, for instance, activation of the JAK2-STAT axis [52], which is similar to the ABCA1 receptor for cholesterol. The pleiotropic effects of GH are coordinated by downstream upregulation of transcription factors such as activator protein 1 (AP-1) [52] that, in turn, triggers a secondary wave on gene regulation to set in motion biological programs for cell proliferation, differentiation, and survival; and such effect has been consistent across many cell types including intestinal cells [53].

The presence of IGF receptor (IGFR) and insulin receptors (InsR) in neonatal enterocytes allow colostral IGF-1, IGF-2, and insulin to work in concert through these receptors, depending on their respective affinity [15]. Because of their similar mode of action through IGFRs and InsR, IGF-1, IGF-2, and insulin produce a similar signal cascade through kinases that is finalized with a change in gene expression [15] similar to GH and cholesterol.

Besides the bioactive compounds described above, colostrum also contains smaller particles called exosomes, which contain various RNA species, including miRNA and lncRNA [14, 49]. The importance of miRNAs in colostrum has been emphasized through several studies, and putative mechanisms have been provided on how these RNA molecules can alter the biology of neonates [49]. Compared to miRNAs, lncRNAs have only now starting to begin to be investigated in bovine colostrum [14]. The significant implication of miRNAs and lncRNAs in colostrum for neonates is the proven absorption of these non-coding RNAs by the small intestine or so-called horizontal gene transfer [54], which is the movement of genetic material between unicellular or multicellular organisms other than vertical transmission from parent to offspring (i.e., reproduction). Once the small intestine absorbs these non-coding RNAs, they can affect the normal translation of endogenous mRNA into proteins, and effectively changing the biology of the offspring [55, 56]. This concept is novel, and there is limited data in bovine neonates testing these effects; it is conceivable that a higher absorption of non-coding RNAs in colostrum will occur soon after calving and before gut closure [57]. In the context of the bovine neonate, these effects need further exploration to understand the impact of miRNAs and lncRNAs on gut health and maturation. For instance, in a combination of intestinal pig cell lines and rodent models, it was observed that miRNAs in porcine milk exosomes significantly promote villus height and crypt depth of the duodenum and jejunum [36, 58]. If the previous effects on gut maturation can be proven in the bovine neonate, this horizontal transfer mechanism of non-coding RNAs could be harnessed amid to deliver optimal non-coding RNAs species in colostrum potentially through maternal nutrition or management during the late gestation.

### Enteric immune development

The importance of gut development and maturation in the neonate goes beyond the mere absorption of nutrients and passive transfer of IgG soon after birth. This importance is rooted in the realization that the gastrointestinal tract is the largest immune organ of the body, and the mucosal immune system is the first line of defense against harmful pathogens [59]. The mucosal immune system allows epithelial cells and microbiome to interact safely, and its development starts in the fetus [60]. The paradigm that the fetus develops within a sterile environment, born bacteria-free, and its first contact with bacteria only occurs after birth has been fundamentally challenged [61]; the presence of microbiota was detected in newborn human meconium [62], amniotic fluid and in umbilical cord blood [63], as well as in placental membrane [64]. Similar to humans, bacteria were detected in newborn calf meconium [65] and bovine uterus [66]. Maternal oral and gut microbiota has been reported to be closely related to placental microbiota and, by extension, to the meconium microbiota, suggesting that maternal microbiota reaches the fetus through the uterus and placental blood vessels. Thus, it is plausible that maternal conditions (e.g., nutrition, stress, and antibiotics) may affect the initial in-utero microbiome colonization and, consequently, the development of the prenatal mucosal immune system. Without a doubt, the particular microbiota profile colonizing the fetal gut will interact with the developing mucosal immune system, and the impact of this interaction in postnatal gut health remains unclear and goes beyond the scope of this review.

The fetus is primarily protected by the innate immune system; however, the phagocytic capacity of fetal neutrophils and macrophages increases during gestation, only to be interrupted near birth by the increase in fetal cortisol [67]. Lymphocytes from the adaptive immune system, develop from hematopoietic stem cells [68]. After being released into the blood, they populate specific locations for further differentiation. The thymus in the case of T cells, and bone marrow or Peyer patches for B cells [68]. All major components of the adaptive immune system are fully developed by 1 month before birth, at which point T cells decline as they populate the lymphoid tissues of the fetus, whereas B cells accumulate across the Peyer patches in the small intestine [59].

In a section above, we commented on the effects of colostrum on intestinal maturation; however, intestinal maturation is not disconnected from the establishment of proper enteric immunity. The bioactive compounds found in colostrum can be seen as mechanisms to procure vital nutrients, not just for calf sustenance but also to procure nutrients to jump-start the enteric immunity. Colostrum contains three main components to provide immunity to the newborn calf: 1) antibodies, 2) cytokines, and 3) immune cells [59]. It is well known that the most important component of colostrum is to provide antibodies to the newborn calf with ~ 55 mg/mL of total IgG. Cytokines, such as IL-6, IL-1β, and TNF-α, delivered via colostrum, are associated with a pro-inflammatory response and will help in recruiting and developing neonatal lymphocytes into the gut while improving phagocytosis and oxidative burst in neutrophils [69]. Simultaneously, anti-inflammatory cytokines, including IL-10, are also present in colostrum, which helps to lessen local inflammatory conditions to allow commensal gut microbiota colonization while maintaining tight junctions [70]. Finally, colostrum delivers leukocytes to the small intestine in similar concentrations to those observed in the peripheral blood, with minor differences. For example, colostrum contains more macrophages (40–50%) and less lymphocytes (22–25%) and neutrophils (25–37%) [71].

The fully developed mucosal immune system can be divided into an outer and inner mucosal layer, epithelium layer, lamina propia, and Peyer patches [59]. Goblet cells are the primary responsible for the formation of the mucous layer by producing mucin and mucous. This mucous secretion creates a highly dense crossed-linked proteoglycan gel in the inner mucous layer, and this coupled with a high concentration of antimicrobial peptides restrict microbiota colonization (i.e., kill zone). The prenatal intestinal population of goblet cells likely increases over the gestation, and by extension, the production of mucosal layer. Compared to term piglets, preterm piglets euthanized at birth had lower intestinal goblet cell density, and the same effect was observed on a similar preterm group euthanized at 11 d of age [72]. The reduction on goblet cells suggests that neonates may take a longer time to produce a protective mucosal layer leaving the intestinal epithelium at risk to be colonized by harmful pathogens. The intestinal epithelium, composed of a single layer of cells, is crucial for preserving gut homeostasis and acts both as a physical barrier and a coordinating hub of immune defense and crosstalk between bacteria and immune cells [73]. Evidence in the bovine neonate indicates that the intestinal epithelium undergoes a great deal of remodeling or maturation soon after birth [74]. This remodeling includes the replacement of vacuolated feta-like intestinal epithelium present at birth by mature intestinal epithelium containing polarized enterocytes. Considering that vacuolated epithelial cells are involved in the transport macromolecules such as IgG in colostrum [75], then gut closure is consistent with the replacement of vacuolated epithelial cells after birth. The repercussions of perinatal stressors, such as heat stress and dystocia, that can delay or alter the natural replacement of vacuolated enterocytes with adult-type enterocytes on neonate gut health remain unknown; thus, this warrants for further scientific interrogation.

### The host side of pathogenic diarrhea

Diarrhea remains as the main disease in preweaned dairy calves and was responsible for over 50% of all deaths in dairy calves born in the U.S. in 2013 [76]. Therefore, diarrhea is single-handedly the biggest threat to the welfare of neonatal dairy calves. Diarrhea in preweaned dairy calves is associated with morbidity, malabsorption, and poor production performance later in life [77]. Preweaning diarrhea is the major factor increasing the age of heifers reaching puberty and lowering milk production and performance in the first lactation [78]. The primary pathogens associated with preweaned dairy calf diarrhea are ETEC, rotavirus, coronavirus, and *Cryptosporidium parvum*. The ETEC typically causes diarrhea in calves during 1–4 d after birth, and its prevalence ranges from 2.6% to 45.1% [16].

A comprehensive review of the pathogenesis of ETEC in neonatal dairy calves was written by Acres [18]. The specific ETEC strain F5 (also designated K99) is the most common cause of neonatal calf diarrhea [77, 79]. Following ingestion, ETEC can colonize the distal portion of the small intestine, which provides a favorable pH environment (~ 6.5) [77]. The ETEC strains expressing F5 (K99) refers to the adhesion antigen (i.e., fimbria), which is pathogenic for calves, lambs, and pigs [80]. The ETEC F5 (K99) utilizes this fimbria initially to adhere to the intestinal epithelium, which is facilitated by coupling of the fimbria and the host receptor. Once attached to the epithelium, the ETEC F5 (K99) will further colonize it and eventually cause diarrhea. The host receptor for the F5 (K99) fimbria in ETEC is a glycolipid ganglioside (i.e., NeuGc-GM3) [79], but other intestinal glycolipid gangliosides (i.e., NeuGc-GM2 and Neu-Gc-GD1a) had been observed to bind F5 (K99) [81]. This lack of specificity suggests that this fimbria-receptor binding is a complex interaction. Currently, there is limited data on the expression of these intestinal glycolipids in neonatal dairy calves. Further research in this area should explore maternal effects (e.g., nutrition, stress, management) or postnatal stressors such as dystocia, fetal cortisol, intestinal maturation that can affect the expression of these glycolipids, leaving the neonate at risk to be easily colonized by ETEC and cause diarrhea.

After binding to the intestinal epithelium, the ETEC will multiply and form microcolonies that will cover the surface of the villi several layers thick before they start releasing the enterotoxin [18]. The enterotoxin, which in the case of ETEC F5 (K99), is a cysteine-rich small peptide that binds to the extracellular portion of the guanylyl cyclase enzyme located on the surface of intestinal epithelial cells. Upon activation, guanylyl cyclase will activate a signal cascade that culminates with the activation of the chloride fibrosis transmembrane conductance regulator (CFTR), promoting excessive Cl^−^ secretion into the intestinal lumen and inhibiting NaCl absorption [19]. This condition causes epithelial cell dehydration, and diarrhea occurs in the animal. Additional mechanisms have been proposed for the enterotoxin mode of action, including regulation of inflammation by promoting arachidonic acid metabolites such as prostaglandins and leukotrienes [80]. In addition to this effect on inflammation, Dubreuil et al. [82] suggested that ETEC enterotoxins cause loss of tight junctions integrity, which could further exacerbate water secretion into the intestinal lumen causing dehydration.

**Fig. 1:**
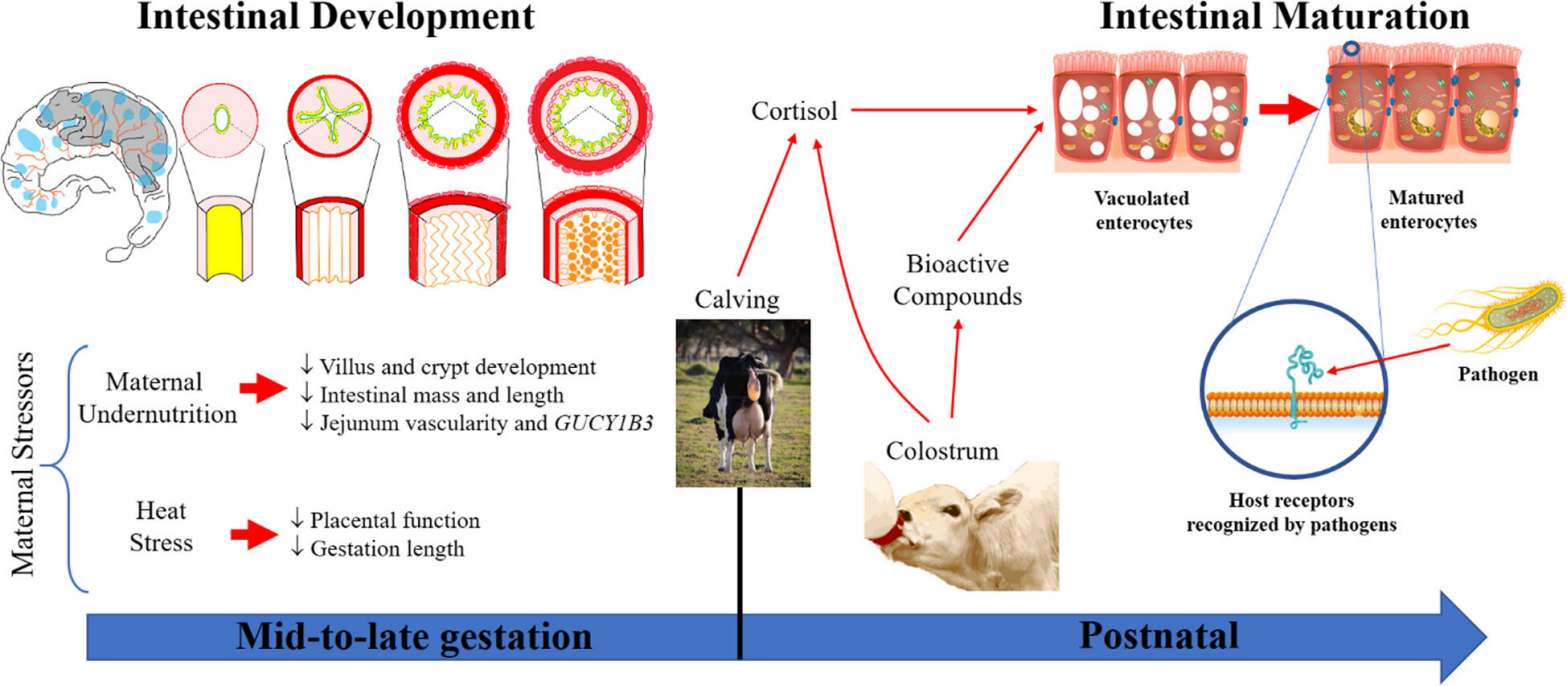
Schematic representation of perinatal stressors and factors that can impact intestinal development and maturation. Before calving maternal nutrition (e.g., undernutrition) can have a detrimental effect on intestinal growth and functionality, including downregulation of soluble guanylate cyclase 1β3 (gene symbol *GUCY1B3*) in the jejunum. Prenatal heat stress may lead to lower placental function and reduced gestation length. The latter can considerably affect the newborn’s ability to ingest valuable nutrients, immunoglobulins, cortisol, and bioactive compounds (e.g., growth factors and hormones) in colostrum, consequently, disrupting the natural intestinal maturation after birth. Intestinal maturation refers to the timely replacement of highly vacuolated enterocytes for more matured and highly functional enterocytes as well as increased crypts depth and cell proliferation. Intestinal diseases in neonatal dairy calves have been associated with morphological and functional immaturity. In this context, little is known about how intestinal immaturity can render neonatal calves vulnerable to pathogen recognition of host receptors (or docking sites), allowing the initial attachment of such pathogens to the intestinal epithelium. For instance, glycolipid gangliosides (e.g., NeuGc-GM3) are host receptors located in the cell membrane epithelium that facilitates the attachment of enterotoxigenic *Escherichia coli* (ETEC). Intestinal development figure adapted from Walton et al. [83]

In the bovine neonate, besides the known route of ETEC enterotoxin leading to efflux of chloride, little is known on the specific transcriptional alterations this enterotoxin can cause in the enterocytes. Furthermore, transcriptional data is a reasonable route to collect biological data on how stress and physiology during the perinatal period can leave the neonate susceptible to ETEC infections and diarrhea. For instance, it remains unknown whether or not maternal effects, colostrum quality, and intestinal maturation can lead to the overexpression of membrane glycolipids receptive to ETEC F5 (K99) and, therefore, increase susceptibility to colonization and enterotoxin modifications (Fig. 1). Compared to the bovine neonate, molecular changes in piglets’ enterocyte in response to ETEC challenge have been investigated. For instance, Niewold et al. [84] and Loos et al. [85] observed a similar upregulation of genes in the intestinal transcriptome related to the innate immune system including extracellular matrix (*MMP1*), cytokines (*IL8*, *IL17A*, *IL1B*), and inflammation (*STAT3*) when piglets were ETEC challenged. From the bovine neonatal standpoint, there is still much to know at the molecular level regarding the ETEC colonization, enterotoxin effects as well as host expression of glycolipids responsible for allowing ETEC to anchor to the intestinal epithelium.

### Novel techniques to understand the neonatal gut biology

A fecal score system is commonly used by researchers and dairy producers to keep track and identify young animals with diarrhea. More sophisticated methods such as microscopy, real-time quantitative-PCR, and bacterial culturing have been used to identifying specific strains of infectious pathogens causing diarrhea. However, from a research standpoint, a common limitation on these analyses, along with the fecal score, is the lack of information on the molecular adaptations the intestinal epithelium undergoes during the onset of diarrhea. In this context, postmortem procedures have been commonly used, and more recently, less invasive alternative methods have been explored by either fecal RNA isolation [86] or via endoscopy biopsy of the colon [87].

The intestinal epithelial cells act as a barrier against pathogens, and the daily shedding of these cells into feces has been used in rodents and humans as a source of RNA to evaluate gene expression in the gastrointestinal tract (GIT) [88]. This method has also been used to find transcriptomic similarities in the GIT between humans and wild primates [89]. Using a similar approach, RNA was isolated from fecal samples that our group refer to as “fecal RNA” from neonatal dairy calves [86]. During this experiment, a typical increase in fecal scores around 2 weeks. after birth was observed, which is a common response in neonatal calves when moving from a passive immunity to an active immunity [59]. Parallel to this increase in fecal scores, it was observed an increase in blood biomarkers associated with inflammation (i.e., IL-6, ceruloplasmin, and haptoglobin) and upregulation in pro-inflammatory genes (i.e., *TLR4*, *TNFA*, *IL8*, and *IL1B*) in fecal RNA. Although this technique is promising, and it has been used by other research groups in rodents [90] and humans [89, 91], we consider that further optimization and validation steps are required in order to improve accuracy and robustness. For instance, the migration of polymorphonuclear leukocytes (PMNL) across the intestinal epithelium is a natural pathological event of many mucosal inflammatory diseases [92]. In order to account for this effect, we evaluate the RNA enrichment from PMNL cells during fecal RNA isolation from the same neonatal dairy calves via RT-qPCR of gene markers for PMNL (i.e., *SELL* and *MPO*) and intestinal epithelial cells (i.e., *KRT8)* [93]. This RT-qPCR analysis included a single standard curve composited of equal amounts of all samples, including cDNA from fecal and PMNL. Results from this study showed greater mRNA expression of *SELL* in PMNL and no expression of *MPO* in fecal RNA, while *KRT8* was greater in fecal RNA. These results suggest a minimal enrichment of immune cells in RNA isolated from fecal samples.

**Fig. 2:**
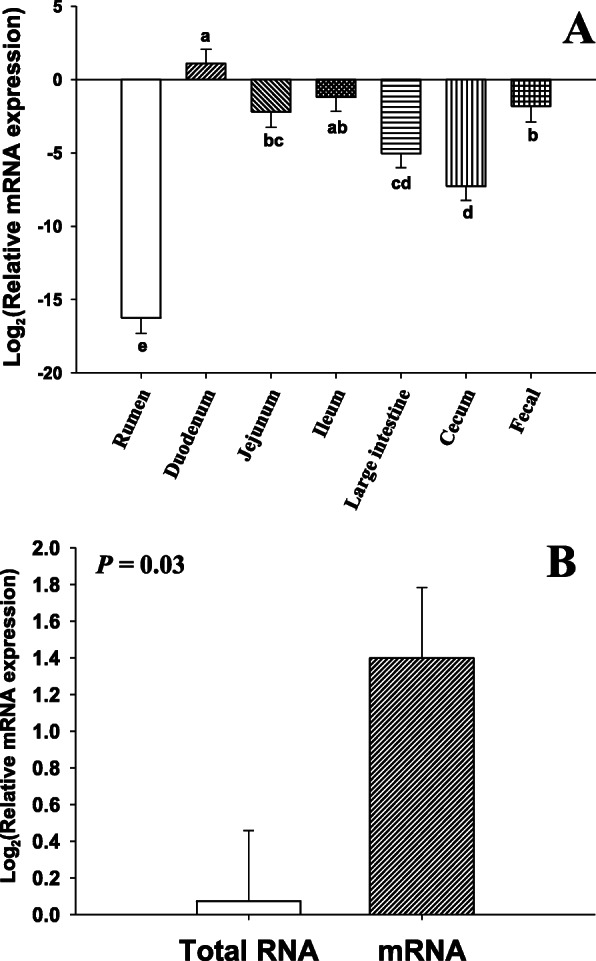
**a** Fatty acid binding protein 2 (*FABP2*) relative mRNA expression in the gastrointestinal tract section, including rumen, duodenum, jejunum, ileum, large intestine, and cecum as well as fecal samples taken simultaneously from eight Jersey dairy calves at 5 weeks of age. Fresh fecal samples were collected prior to euthanasia, and gastrointestinal samples were collected as tissue specimens, and all samples were flash-frozen immediately in liquid N. RNA extraction, and RT-qPCR analysis was performed according to protocols detailed in Rosa et al. [86]. The mRNA expression data were analyzed using ANOVA with type of tissue (e.g., fecal, rumen, etc.) as the fixed effect and calf as the random effect. **b**) Relative mRNA expression of keratin 8 (*KRT8*) in total RNA and mRNA isolated from the same fecal samples (*n* = 8). Total RNA extraction was performed according to (Rosa et al., [86]), and mRNA was isolated from the same total RNA samples (details in Suppl. Materials). A comparison between total RNA and mRNA was performed using similar protocols as (Rosa and Osorio, [93]), where a single standard curve was constructed with equal amounts of cDNA samples from total RNA and mRNA. The mRNA expression data were analyzed using ANOVA with type of RNA (i.e., total RNA and mRNA) as the fixed effect and calf as the random effect. Data in Panel A and B were log-transformed prior to statistical analysis with the Proc Mixed procedure of SAS 9.4. ^a-e^Superscripts denoting a statistical significance at *P* ≤ 0.05

Fatty acid binding protein 2 (*FABP2*) gene is highly expressed in small intestine, and it has been identified as a specific marker for intestinal epithelium in humans [94]. In non-ruminants, mRNA *FABP2* expression in the small intestine of pigs was associated with intestinal permeability [95], and loss of enterocytes was correlated with a downregulation of *FABP2* expression in the jejunal mucosa of broiler chickens [96]. In order to establish associations between fecal RNA and the GIT, we deemed necessary to interrogate the mRNA transcription of *FABP2* in GIT tissues (i.e., rumen, duodenum, jejunum, ileum, large intestine, and cecum) and compare these with fecal RNA. Results from this analysis indicate that mRNA transcription in fecal RNA may resemble those in the small intestine, particularly jejunum and ileum (Fig. 2a [97]).

**Fig. 3:**
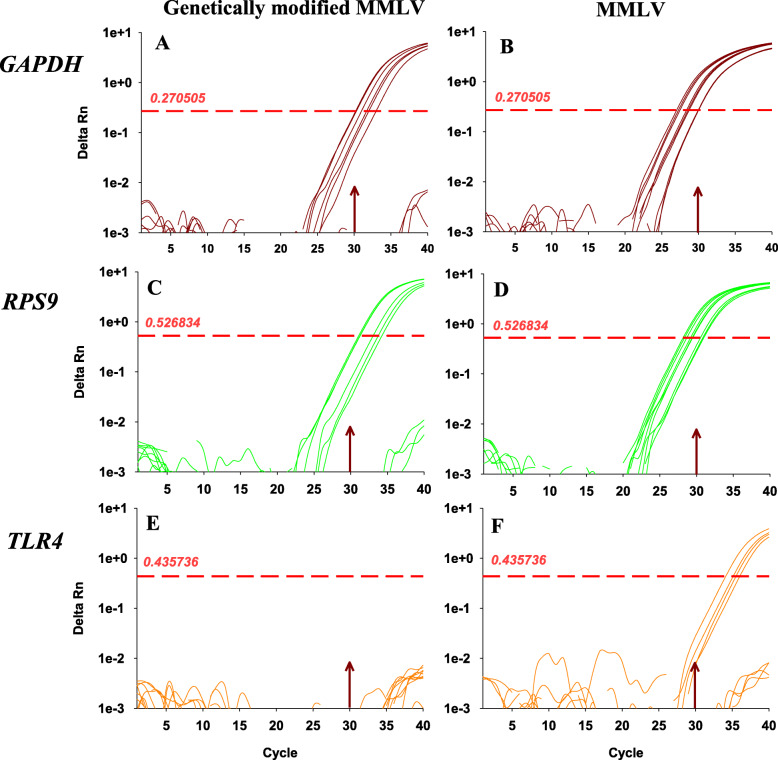
Amplification plots from RT-qPCR analysis on total RNA from fecal samples for glyceraldehyde 3-phosphate dehydrogenase (*GAPDH*), ribosomal protein S9 (*RPS9*), toll-like receptor 4 (*TLR4*). The reverse transcriptase Moloney murine leukemia virus (MMLV) used in this test was SuperScript IV (Cat# 18090050; Invitrogen, Carlsbad, CA, USA), while the genetically modified MMLV was RevertAid (Cat# EP0442; Thermo Scientific, MA, USA). Three fecal samples from different calves were used to isolate total RNA, which was, in turn, used to synthesize cDNA using equal amounts of either reverse transcriptase (i.e., MMLV and genetically modified MMLV). Dashed line in red indicates the threshold with the actual value in red for each gene. Arrow denotes the 30th amplification cycle. Additional protocol information can be found in the Suppl. Materials

**Fig. 4:**
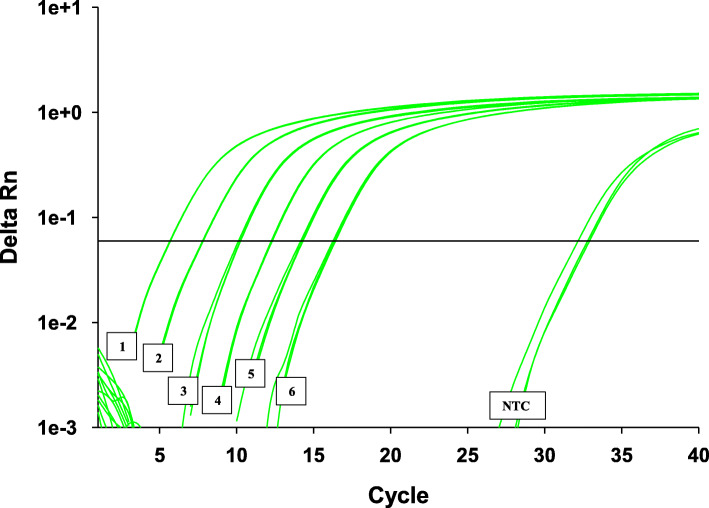
Amplification plot of a known concentration of *GAPDH* (gBlock gene fragment; Integrated DNA Technologies) ranging from 0.2 μg to 0.0002 μg (or 200 pg) in a 1:4 serial dilution. The quantitative PCR reaction was performed in a QuantStudio 6 Flex Real-Time PCR System (Applied Biosystems) using FAM as the reporter dye, NFQ-MGB as the quencher, and ROX as the passive reference. The RT-qPCR assay was performed using a pre-designed PrimeTime® Mini qPCR Probe from IDT Integrated DNA Technologies, which consists of a *GAPDH* primer pair and 5′ nuclease probe. The RT-qPCR reaction was performed using 10 μL of qPCR mixture containing 4 μL cDNA template, 5 μL of the PrimeTime gene expression master mix (Cat# 1055770, IDT Integrated DNA Technologies), 0.5 μL of PrimeTime®qPCR Assay (Premixed primers and probe), 0.5 μL nuclease-free water in a MicroAmp Optical 384-well reaction plate (Applied Biosystems). The qPCR reactions were performed using the following conditions: 3 min at 95 °C, 40 cycles of 15 s at 95 °C, and 1 min annealing at 60 °C. Actual Cts, standard curve dilutions, and *GAPDH* concentrations are presented in Suppl. Materials Table 1

Robust data can be generated with the application of high-throughput molecular techniques such as RNAseq to study the transcriptome in RNA isolated from fecal samples, which have been consistently reported across different species mouse [98], infants [91], wild primates [89]. However, when analyzing specific genes in fecal RNA through RT-qPCR has been proven challenging. This partially due to low consistency on early protocols using fecal RNA for gene expression analysis [90, 99], and a lack of proper validation of internal control genes that can be consistently used to stabilize transcriptional variation across samples. The study of intestinal gene expression in bovine neonates will substantially benefit from an improved RT-qPCR protocol adjusted to this type of sample; this funded on the inherited dynamic environment of the GIT. Thus, RT-qPCR is more suited than omics methods, to investigate specific genes in a longitudinal format, for instance, during intestinal maturation, diarrhea events, specific pathogen infections (e.g., ETEC), or heat stress. The nuance we have observed in our laboratory when optimizing an RT-qPCR protocol for fecal RNA are:

- Isolation of mRNA from total fecal RNA may improve the sensitivity of the RT-qPCR analysis since we have previously [86] observed that total fecal RNA contains a significant amount of bacterial RNA and this has been confirmed via RNA-seq data in primates [89]. In Fig. 2b, it is evident a greater expression of *KRT8* in mRNA samples than total fecal RNA.
- Selecting an adequate reverse transcriptase enzyme for cDNA synthesis can have a significant impact on RT-qPCR data, as reported by Sieber et al. [100]. In our case, we observed a significant improvement in the amplification of genes with a known high (e.g., *GAPDH* and *RPS9*) and low (e.g., *TLR4*) expression in fecal RNA when using a reverse transcriptase from Moloney murine leukemia virus (MMLV) in comparison to a genetically modified MMLV (Fig. 3).
- Understanding the sensitivity of an assay or analysis is essential. In a standard RT-qPCR, a standard curve is utilized in order to estimate the transcripts concentration for a particular gene across several dilutions; hence, relative mRNA expression. In this test, we introduced a known gene, *GAPDH* (gBlock gene fragment; Integrated DNA Technologies), with a known concentration (i.e., 0.2 μg) into cDNA from fecal RNA samples and then perform several dilutions, similarly to a standard curve. Then, we utilized TaqMan gene expression assay with specific primers and probe targeting the known gBlock sequence of *GAPDH*. Results from this test suggest that RT-qPCR has a high sensitivity for detection of genes from concentrations of 0.2 μg to 0.0002 μg (200 pg), yet, it is evident that RT-qPCR could still detect genes with a much lower expression (Fig. 4).

### Future implications for gut health in neonatal dairy calves

Fetal programming research in livestock animals has paved the way for understanding the maternal influence in progeny’s survival rate, growth performance, feed efficiency, among other parameters pertinent to production systems for livestock animals. Gut health has become a central topic of research due to its many ramifications, including immunocompetence, feed efficiency, gut-brain axis, and microbiome. Although, the latter has provided us with a vast amount of new information on the dynamics of microbial communities in the neonatal gut, this is one side of host-microbiome interaction. Therefore, newer technologies and bioinformatics tools should focus on recovering data from both the host and the microbiome to provide a more holistic analysis in terms of prenatal intestinal development or postnatal intestinal maturation. Indeed, the marriage of fundamental fetal programming research with more holistic measurements of gut health will be enlightening, and the identification of key responses on intestinal development or maturation to changes in diet, management, physiological state, and genetics may well lead to more accurate recommendations for the pregnant dairy cow or the newborn calf.

PCR-based molecular technologies have become more ubiquitous in animal science research in the last decade, and eventually some of these procedures and protocols have started to be oriented towards a more farm-based application. For instance, PCR-based assays for rapid detection have been developed to identify mastitis pathogens in dairy cows or influenza A in growing pigs. The potential knowledge to be gain from GIT gene expression data could lead to identify molecular markers for control points that make animals vulnerable to enteropathogens such as specific glycolipid gangliosides allowing the anchoring of ETEC to the intestinal epithelium. Then, it could be plausible to generate PCR-base assays for on-farm identification of newborn calves at risk of diarrhea based on molecular markers (e.g., glycolipid gangliosides) found in fecal RNA.

The available data support a stronger influence of maternal nutrition during mid- to late gestation on fetal intestinal development. Prior to this time, pregnant cows experience lower nutrient requirements, especially during late lactation and early dry period, leading to a potential overconditioning. Therefore, controlling energy intake or targeting for a moderate BCS at calving for dairy cows has become a common practice among dairy farms. Without a doubt, nutritional interventions during the dry period or late gestation have an impact on birth weight and intestinal development. This impact, as reported in nutrient restriction experiments, can imprintan “intestinal programming” that seems to allow the offspring to compensate for prenatal nutritional insults. However, the implications of such alterations through life in the newborn are yet to be understood. The intent of this review has been to provide a framework on the current knowledge on the maternal influence on perinatal gut physiology in dairy calves and how this relates to the host-pathogen interaction, but much work remains to be done. Key remaining questions regarding perinatal gut physiology in dairy calves include the following:

- How much does prenatal intestinal underdevelopment affects the adequate colostrum IgG absorption at birth and further intestinal maturation?
- To what extent does intestinal underdevelopment or maturation explains (or predict) survival rate, incidence of diarrhea, feed efficiency, residual feed intake? Then, if any of these are elucidated, what corrective approaches can be taken?
- Controlled-energy diets fed during the dry period of dairy cows are commonly formulated (~ 100% requirements) for a less aggressive restriction than the IUGR model. Then, what is the extent of the impact of a controlled-energy diet during late gestation on fetal intestinal development?
- What is the relationship between overconditioned dams at calving and offspring’s intestinal development? What is the long-term effect on intestinal programming in dairy calves born to overconditioned dams? If such programming is negative in terms of health and performance, how can we revert such programming via postnatal nutrition and management?
- Given the need of enteropathogens to anchor themselves to the intestinal epithelium via molecular docking sites (e.g., glycolipid gangliosides allowing ETEC infection), how much of the incidence of neonatal diarrhea can be attributed to overexpression of specific molecular docking sites rendering neonates vulnerable to enteropathogens? If such overexpression exist, could this be linked to any of the perinatal stressors discussed previously?
- How GIT gene expression data may help explain some of previous questions? How reliable are noninvasive methods such as fecal RNA in the context of bovine neonates? Then, how can we combine molecular data between the host and pathogens to provide a more holistic approach to neonatal diarrhea?

## Conclusions

The cumulative evidence that perinatal events have long-lasting ripple effects through the life of livestock animals should impact future nutritional and management recommendations at the farm level. The continuous margin of morbidity and death in preweaning dairy calves suggests that there is still room for improvements in nutrition and management in dairy farms. Data suggest that minimizing maternal stressors, such as malnutrition and heath stress, can significantly improve fetal development, specifically intestinal ontogeny and development. This being fundamental not only for the acquisition of passive immunity at birth but also for the absorption of bioactive compounds necessary for postnatal intestinal maturation. Residual effects of prenatal intestinal underdevelopment can still be detected in the offspring over a year old, which underscores the potential impact of prenatal stressors over the lifetime of livestock animals. This reduction in prenatal gut development can be the product of reduced exposure to the cortisol surge during the days prior to calving. In addition, maternal nutrition and fine-tuning of key components in the diet might offer solutions to improve adequate prenatal intestinal development. The recent change in paradigm regarding early microbiota colonization of the fetal gut has added a whole new layer of complexity on prenatal intestinal development and the mucosal immune system. The fact that buccal microbiota has been correlated with fetal gut microbiota, strongly suggests a maternal-placental-fetal axis of colonization during pregnancy. This begs the questions if future management recommendations on transition cows should include manipulation of rumen or lower gastrointestinal tract microbiota to stimulate the early fetal gut colonization of beneficial microbes that can improve intestinal development, mucosal immune system, and colostral IgG absorption. The fecal RNA approach proposed here can provide invaluable knowledge on these effects and others such enteropathogenesis from the host side of the host-pathogen interactions. In-depth knowledge of the neonatal intestinal biology rooted in molecular data and coupled with functional parameters and phenotypical data will help promote bovine neonate well-being in the future while improving profitability in dairy farms.

## Supplementary information

**Additional file 1.**

## Data Availability

The data presented in this document are available from the corresponding author on reasonable request.

## Abbreviations

miRNAs: microRNAs
lncRNAs: Long non-codings RNAs
AA: Amino acids
IUGR: Intrauterine growth retardation
IGF: Insulin-like growth factors
ETEC: Enterotoxigenic *Escherichia coli*
GUCY1B3: Soluble guanylate cyclase 1 β3
SLC5A1: Solute carrier family 5 member 1
CD36: Cluster of differentiation 36
CCK: Cholecystokinin
HPA: Hypothalamus-pituitary-adrenocortical
DCAD: Dietary cation-anion difference
GH: Growth hormone
MAPK: Mitogen-activated protein kinase
JAK: Janus kinase
STATs: Signal transducer and activators of transcription
GHR: Growth hormone receptor
ABCA1: ATP-binding cassette transporter A1
AP-1: Activator protein 1
IGFR: Insulin-like growth factor receptor
InsR: Insulin receptors
IGF-1: Insulin-like growth factor 1
IGF-2: Insulin-like growth factor 2
IgG: Immunoglobulin G
IL-6: Interleukin 6
IL-1β: Interleukin 1β
TNF-α: Tumor necrosis factor α
IL-10: Interleukin 10
MMP1: Matrix metalloproteinase 1
IL8: Interleukin 8
IL17A: Interleukin 17A
STAT3: Signal transducer and activator of transcription 3
PCR: Polymerase chain reaction
GIT: Gastrointestinal tract
TLR4: Toll-like receptor 4
PMNL: Polymorphonuclear leukocytes
RT-qPCR: Real-time quantitative polymerase chain reaction
SELL: L-selectin
MPO: Myeloperoxidase
KRT8: Cytokeratin 8
FABP2: Fatty acid binding protein 2
GAPDH: Glyceraldehyde 3-phosphate dehydrogenase
RPS9: Ribosomal protein S9
MMLV: Moloney murine leukemia virus
