# Supplementary material for: Gut health, stress, and immunity in neonatal dairy calves: the host side of host-pathogen interactions
Source: J Anim Sci Biotechnol. 2020 Nov 9;11:105. doi: 10.1186/s40104-020-00509-3 (PMC7649058; doi:10.1186/s40104-020-00509-3)
Supplement: Supplementary file 1 — Additional file 1. [file 40104_2020_509_MOESM1_ESM.docx]

**SUPPLEMENTAL MATERIALS**

**Protocol for mRNA isolation from total RNA**

First, fecal samples were collected from the animal by rectum stimulation and immediately flash-frozen in liquid nitrogen [1]. Then, the total RNA isolation from fecal samples was performed, starting with 400 mg of fecal material placed in a 15-mL RNase/DNase free tube with 1.5 mL of Trizol (Invitrogen Corp., Carlsbad, CA) at 4 °C. Samples were disrupted thoroughly using a tissue homogenizer until samples are visibly homogenized. All samples are kept on ice during the process. Subsequently, 240 µL of Phenol-Chloroform are added to the lysate solution (samples immersed in trizol) in order to isolate the RNA from the organic phase. Tubes are vigorously shaken by hand for 15 s and incubated at 4 °C (on ice) for 10 min. After centrifugation at 14,000 ×*g* for 15 min at 4°C, the upper phase supernatant is transferred in a new 2.0 mL RNase/DNase free microtube. Then, the total RNA isolation is purified using the Qiagen silica-membrane spin-columns for the selection of high-quality RNA. The steps for RNA purification and clean up are performed following the procedure recommended by Qiagen (RNeasyPlus Mini Kit; Cat. No. 74134). The final RNA is collected in 50 µL RNase-free water. The total RNA quantity (ng/µL) and purity (OD260/280) is determined using Nanodrop instrument [2].

In a second step, 75 µg of total RNA is used as starting material for the mRNA purification. The total RNA samples are heated to 65°C for 2 min in order to disrupt secondary structures. All samples should be kept on ice immediately after incubation. For the isolation of highly purified mRNA from total RNA the Dynabeads® Oligo dT_25_ (Ambion, Cat#61006) is used. The principle for that relies on base-pairing between the poly A residues at the 3’ end of mRNA and the oligo (dT)25 residues covalently coupled to the surface of the Dynabeads®. Other RNA species lacking a poly A tail will not hybridize to the beads and are readily washed away. Based on that, 0.5 mg (100 µL) of Dynabeads® is transferred to a 2.0-mL RNase/DNase free microtube, and through magnetic isolation, the Dynabeads® are resuspended and calibrated with 100 µL of binding buffer. The Dynabeads®/binding buffer solution will allow the hybridization between total RNA and the oligo (dT)25. For the annealing between mRNA present in each sample to the oligo (dT)25 present on the beads an incubation of the Dynabeads®/binding buffer solution with the total RNA is required for 10 min at room temperature. Such incubation should be performed in roller or mixer that allows the samples to be thoroughly mixed. Then, through magnetic isolation, the mRNA-bead complex is washed, and 50 µL of 10 mmol/L Tris-HCl is added in each mRNA-bead complex solution and heated to 70°C for 2 min in order to elute the mRNA from the bead solution. Immediately, after this incubation, the samples are placed in the magnet for the final separation. The beads would have migrated to the tube wall, and the purified mRNA eluted at the bottom of the tube. The final mRNA solution can be measured in Nanodrop instrument and stored in -80°C freezer until further analysis.

**Reverse transcriptase comparison**

Total RNA from fecal samples was isolated following the same procedures described above in the Protocol for mRNA isolation from total RNA. The cDNA synthesis was performed simultaneously from the same fecal samples using either an MMLV reversed transcriptase enzyme (SuperScript IV; Cat# 18090050; Invitrogen, Carlsbad, CA, USA) or a genetically modified MMLV variant (RevertAid; Cat# EP0442; Thermo Scientific, MA, USA). Each cDNA was synthesized by reverse transcription using 400 ng RNA, 1 µL Random Primers (Cat# 48190-011, Invitrogen, Carlsbad, CA, USA), and 5 µL DNase/RNase-free water (Cat# 10977-015, Life Technologies, Grand Island, NY). The mixture was incubated at 65°C for 5 min and kept on ice for 3 min. A total of 9 µL of Master Mix composed of 1 µg dT18 (Invitrogen, Carlsbad, CA, USA), 2 µL 10 mmol/L dNTP mix (Cat# 18427-013, Invitrogen, Carlsbad, CA, USA), 4 µL 5 × Reaction Buffer, 0.25 µL RevertAid Transcriptase (Superscript IV or RevertAid), 0.125 µL Rnase Inhibitor (40 U/µL; Thermofisher; Cat. No. EO0382), and 1.625 µL DNase/RNase-free water were added to each sample. The second step of the cDNA synthesis reaction was performed as follows: 25°C for 5 min, 42°C for 60 min, and 70°C for 5 min. The synthesized cDNA was then diluted 1:3 with DNase/RNase-free water. The qPCR reaction was performed in a QuantStudio 6 Flex Real-Time PCR System (Applied Biosystems) in MicroAmp® Optical 384-well Reaction Plate (Applied Biosystems, USA) as described in Bionaz and Loor [3]. The qPCR reactions were performed using the following conditions: 2 min at 50°C, 10 min at 95°C, 40 cycles of 15 s at 95°C, and 1 min annealing at 60°C.

**Table 1.** Detection of *GAPDH* in the standard curve points (STD 1 – STD 6) of fecal RNA isolated from dairy calves.

|  | CT^1^ | STD dilution^2^ | *GAPDH*, µg^3^ |
| --- | --- | --- | --- |
| NTC^4^ | 32.57 | - | - |
| STD 1 | 5.66 | 1 | 0.2 |
| STD 2 | 7.79 | 0.25 | 0.05 |
| STD 3 | 10.13 | 0.0625 | 0.0125 |
| STD 4 | 12.21 | 0.0156 | 0.0031 |
| STD 5 | 14.19 | 0.0039 | 0.0008 |
| STD 6 | 16.30 | 0.0010 | 0.0002 |

^1^CT = mean cycle threshold, which is defined as the number of cycles required for the fluorescent signal to cross the threshold; ^2^Relative standard curve 6-point of fecal RNA were diluted with 4 fold dilution per point 1:4; ^3^*GAPDH* (glyceraldehyde 3-phosphate dehydrogenase) concentration added in each STD sample; ^4^non-template control (no STD added).

**REFERENCES**

1. Rosa F, Busato S, Avaroma FC, Linville K, Trevisi E, Osorio JS, et al. Transcriptional changes detected in fecal RNA of neonatal dairy calves undergoing a mild diarrhea are associated with inflammatory biomarkers. PLOS ONE. 2018;13(1):e0191599.

2. Rosa F, Osorio JS. Short communication: Comparative gene expression analysis on the enrichment of polymorphonuclear leukocytes and gastrointestinal epithelial cells in fecal RNA from nondiarrheic neonatal dairy calves. Journal of Dairy Science. 2019;102(8):7464-8.

3. Bionaz M, Loor JJ. Identification of reference genes for quantitative real-time PCR in the bovine mammary gland during the lactation cycle. Physiological genomics. 2007;29(3):312-9.
